# 

**Published:** 1899-11

**Authors:** 


MINOR SURGERY AND BANDAGING. By Henry R. Wharton, M. D., Demonstrator of Surgery in the University of Pennsylvania. New (4th) edition. In one 12mo. volume of 594 pages, with 502 engravings, many being photographic. Cloth, $3.00, net. Lea Brothers & Co., Philadelphia and New York.

This has been a standard work for some time, and has its right to live well established among medical schools throughout the country. This, the fourth edition, has been brought up to date and includes much new matter. A chapter on surgical bacteriology, and also one on operative surgery upon the cadaver, have been added, which makes the edition all that could be desired. The style is free from ambiguity, the numerous cuts illustrate the text without confusing the student, and the general arrangement of the subject matter is admirable throughout the book. B.

